# Efficacy and Safety of Topical Corticosteroids for Management of Oral Chronic Graft versus Host Disease

**DOI:** 10.1155/2017/1908768

**Published:** 2017-07-02

**Authors:** Basma Abdelaleem Elsaadany, Eman Magdy Ahmed, Sana Maher Hasan Aghbary

**Affiliations:** ^1^Faculty of Dentistry, Cairo University, Giza, Egypt; ^2^Faculty of Oral and Dental Medicine, Beni Suef University, Beni Suef, Egypt; ^3^Faculty of Dentistry, Aden University, Aden, Yemen

## Abstract

**Background:**

Oral chronic graft versus host disease (cGVHD) is a major complication in transplantation community, a problem that can be addressed with topical intervention. Topical corticosteroids are the first line of treatment although the choice remains challenging as none of the available treatments is supported by strong clinical evidence.

**Objective:**

This systematic review aims to determine the clinical efficacy and safety of topical corticosteroids for the management of the mucosal alterations of oral cGVHD.

**Data Sources:**

Electronic search of different databases was conducted: PubMed, Cochrane library, Grey literature, WHO, and clinical trials.gov for clinical trial registration as well as hand search in the references of relevant articles up to November 2016.

**Data Extraction:**

Extracted pieces of information were intervention, population, sample sizes, and outcomes.

**Data Synthesis:**

Six studies were included: 2 randomized clinical trials (RCTs), 3 cohort studies, and 1 pre-post clinical trial.

**Results:**

There is a limited evidence concerning clinical efficacy of topical corticosteroids. Clobetasol, dexamethasone, and budesonide were the topical corticosteroid of choice. The highest level of evidence score was given to clobetasol followed by budesonide with a lower evidence level.

**Conclusion:**

All three topical corticosteroid preparations are effective for management of oral chronic GVHD with minimal easily avoided side effects.

## 1. Introduction

Graft versus host disease (GVHD) is a multisystem immunologically mediated disease that has been accused as major complication in allogeneic transplantation process. The disease affects up to half of all hematopoietic stem cell transplant (HSCT) patients [1]. GVHD usually affects the skin, mouth, eyes, gastrointestinal tract (GIT), and liver but other systems such as the lungs, joints, and genitourinary tract may also be involved with the oral cavity being the second most commonly involved organ system, after skin [2]. Oral chronic GVHD (oral cGVHD) is presented as generalized mucosal erythema, erosions, ulcerations, white striae, or papules resembling oral lichen planus and can also develop oral mucoceles. Patients may complain as well from xerostomia and pain [1, 3]. The oral involvement may be the only presentation of the condition or a part of multisystem involvement. Symptomatic management with systemic therapy and topical treatment alone or in combination are the choices to provide local palliation. However, clinically oral cGVHD develops during reduction of systemically administered immunosuppressant after HSCT [3]. Topical agents in oral cGVHD reduce doses or accelerate healing when used with systemic immunosuppressant therapy minimizing their side effects [4].

Usually topical steroids are used to treat oral cGVHD; although they are not specifically approved for oral cGVHD, they have been adopted for use in oral cGVHD based on their well accepted use for other oral mucosal conditions, particularly for oral lichen planus [1].

Budesonide is a synthetic glucocorticoid. Recently it has been used with high efficiency both as local and systemic treatment. The drug has renowned safety profile and has successfully been used topically to treat different oral lesions of cGVHD [5].

Clobetasol is a super potent topical corticosteroid with remarkable results in management of mucocutaneous lesions of cGVHD. The drug has a limited bioavailability providing less systemic effect [6].

Dexamethasone is a prednisolone analogue 25 times more potent with longer duration of action compared to hydrocortisone [13]. Oral lesions due to cCVHD respond dramatically to topical dexamethasone application [10].

This systematic review aims to determine the clinical efficacy and safety of topical corticosteroids for the management of the mucosal manifestations of oral cGVHD.

## 2. Methodology

**Figure 1:**
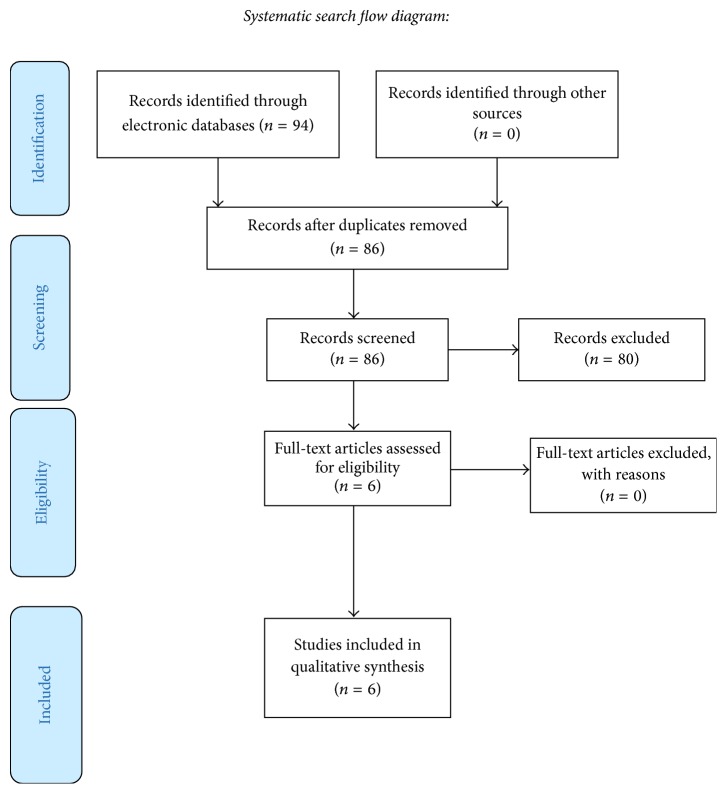
Flow diagram of article selection.

We conducted an electronic search of the following databases PubMed, Cochrane Central Register of controlled clinical trials, WHO clinical trial registration, and Grey literature up to November 2016, based on the search strategy developed for PubMed using the key words “oral”, “chronic”, “graft versus host disease”, and “topical corticosteroids” but revised appropriately for each database and manual searching through the central library of Cairo University, library of Faculty of Dentistry, and the central library of the Egyptian National Institute of Cancer. Also, the bibliographies of included papers and relevant review articles were checked for studies not identified by the search strategies above (see Figure 1).

We included all clinical trials using any form, dose, or concentration of topical corticosteroid preparation for management of symptomatic mucosal lesion of oral cGVHD. We excluded any article with additional systemic therapy other than anti-GVHD medications or treatment with another concomitant topical preparation during the 12 weeks before the trial except for prophylaxis against infections (e.g., antifungal and antiviral agents). Critical outcomes measured are subjective symptoms (pain), clinical improvement (in term of mucositis scale), time to maximum improvement, relapse, and side effects. The authors independently evaluated each included study using the Cochrane risk of bias assessment tool for RCT [5] and SIGN checklist for cohort study [6]. The evidence level of the included studies was assessed using the Oxford CEBM “levels of evidence” [13].

Data extraction form has been generated by the authors to collect all the data items specified in the review synthesis strategy to address the review questions. Any conflict between authors was solved by a methodological expert. Extracted information about the studies (i.e., intervention, population, context, sample sizes, outcomes, and study quality) will be tabulated in a manner consistent with the review question.

## 3. Results

The electronic literature search produced 94 articles; after removal of duplicates 84 underwent screening for title and abstract and 6 met the inclusion criteria (Table 1). No more eligible studies were identified by hand search. The evidence table includes study design, interventions, outcomes (pain reduction, clinical score, time to maximum improvement, relapse, and adverse effects), level of evidence, and grade of recommendation (see Table 2). From the 6 included studies in (Table 1), budesonide, dexamethasone, and clobetasol were the topical steroid agents evaluated in included studies. Of the reviewed studies, there were 2 RCTs [7, 10], 3 cohort studies [8, 9, 11], and 1 pre-post clinical trial [12].

## 4. Dexamethasone

In 2004 there was a retrospective cohort study [11] in which 16 patients with oral cGVHD were enrolled, using 0.01% of dexamethasone sol. as oral rinse for 28 days. The daily dose however was not clearly reported. With a level of evidence evaluated as 4 and Grade C, the results showed that 68.7% of patients reported symptomatic improvement. Median pain visual analogue scale (VAS) difference and side effects of the treatment were not reported.

The authors did not also report the severity of the oral cGVHD at the beginning of the study.

In 2013, a prospective cohort study [8] used the same drug form and concentration also, for 28 days on 24 patients given as 5–10 mL oral rinse 3–6 times per day. The level of evidence of their study was evaluated as 2b graded B. According to severity of oral involvement at baseline 20.8% of patients presented with mild symptoms, 70.8% with moderate symptoms, and 8.3% with severe symptoms. The results of their study showed 27.2% average reduction in modified oral mucositis rating scale (mOMRS), 29.1% of patients reported symptomatic improvement with the topical treatment, and average VAS reduction was 28.5%. The study reported 16.6% of patients complained from side effects, two cases with burning sensation, one with taste alteration, and one with fungal infection. In consistence in 2014 a randomized clinical trial [7] used the same drug, same form and concentration, on 18 patients for 28 days. The level of evidence was 1b graded A. the results showed 50% average reduction in mOMRS, 33% of patients reported symptomatic improvement with the topical treatment, and average VAS reduction was 29.7%. However, the study did not report the severity of the condition at the baseline. Concurrent prophylactic antifungal treatment was prescribed. The study reported 5.5% of patients complaining from possible side effects presented as one case with burning sensation and no reported fungal infections.

## 5. Clobetasol

Only one randomized controlled clinical trial [7] reported the use of 0.05% clobetasol oral rinse, level of evidence evaluated as 1b graded A. The study was conducted on 14 patients with a daily dose of 5 mL three times for 28 days. The results showed 50% average reduction in mOMRS, 85% of patients reported symptomatic improvement with the topical treatment, and average VAS reduction was 44.6%. One case (7% of patients) with burning mouth was reported as possible side effect. Concurrent prophylactic antifungal treatment was prescribed.

## 6. Budesonide

In 2003, a pre-post treatment clinical trial [12] was conducted using 0.06% budesonide oral rinse on 12 patients for 1 to 6 months. The evidence level was evaluated as 4 graded C. The study reported mean time for maximum improvement to be 54 days. With unclear results of mOMRS, the study reported that 100% of patients showed clinical improvement. One case (8%) complained from burning mouth.

In 2007, a retrospective cohort study [9] evaluated topical budesonide along with systemic treatment. They used 0.03% budesonide sol. as oral rinse for 28 days; evidence level was evaluated as 4 graded B. The results showed 66.5% average reduction in mOMRS, 83.3% of patients reported symptomatic improvement with the topical treatment, and average VAS reduction was 54.4%. The study reported one case with burning mouth and one with HSV infection representing 16.6% of patients. The severity of oral involvement at baseline was 25% mild symptoms, 41.6% moderate symptoms, and 33.3% severe symptoms. No concurrent antifungal prophylaxis was used.

Budesonide was investigated again in 2013, but in a prospective cohort study [8] with evidence level 2b graded B, in which they used 0.03% budesonide sol. as oral rinse on 26 patients with oral cGVHD for 28 days. The results of their study showed 50% average reduction in mOMRS, 53.8% of patients reported symptomatic improvement with the topical treatment, and average VAS reduction was 37.5%. The study reported 19% of patients complaining from side effects presented as one case with taste alteration and four with fungal infection. The severity of oral involvement at baseline was 30.8% mild symptoms, 53.8% moderate symptoms, and 15.4% severe symptoms. No antifungal prophylaxis was used throughout the study time.

A pilot randomized clinical trial [10] used the same form and concentration of budesonide different daily doses on 18 patients for 60 days and up to 6 months. The evidence level of this study was 2b graded B, mean time for maximum improvement was 45.5 days, average reduction in mOMRS was 70%, 70% of patients reported symptomatic improvement, and average VAS reduction was 70%. Also, 50% of patients complained from a possible side effects including cheilitis, esophagitis, fungal infection, and taste alteration. A concurrent prophylactic antifungal, antibacterial, and antiviral drug were prescribed.

Regarding the clinical results and concerning the level of evidence and grading, the highest level of evidence score was given to Noce et al. [7] for their RCT stating efficient clinical results for topical clobetasol. This is followed by Park et al. [8] in the good quality prospective cohort study and the pilot RCT of Elad et al. [10] both reporting a promising clinical results of budesonide; the results of those studies enforced the week evidence from Sari et al. [9] and Elad et al. [12].

## 7. Discussion

Six clinical trials were included in this systematic review of evidence evaluating the efficacy and safety of three different topical corticosteroids in the management of oral cGVHD. There was a heterogeneity regarding the trial outcomes and outcome assessment and the mixed study design hindered gathering the studies for statistical analysis. It is acknowledged for most practitioners that topical corticosteroids are the first line of treatment for oral cGVHD [1, 4, 14]. Upon evaluation of the available evidence we agree on the net benefit of topical corticosteroids with a recommendation to “probably” the use of topical steroid is effective in management of signs and symptoms of oral mucosal GVHD with minimal easily avoided side effects. That is because of uncertainty about the quality of the available evidence.

Considering safety of topical corticosteroids, the results demonstrated that oral fungal infection is the most common side effect. However, this problem is strictly governed by the use of antifungal prophylaxis during treatment. Treatment regimen with concurrent prophylactic treatment ended up with no fungal infection [7, 10]. On the other hand oral fungal infection was reported in treatment regimen where no antifungal prophylaxis was added [8, 9].

Uncertainty about the quality of the available evidence is mainly attributed to shortage of high quality RCTs, small sample size, methodological limitations, and inconsistency across studies.

Inconsistency refers to the dissimilarity of estimates of effect across studies.

Lack of randomized clinical trial is a major limitation concerning judging the efficacy and safety of a defined intervention. The low number of clinical trials on this topic and the small sample size notably found in all studies could be attributed to the nature of the disease and practical difficulties concerning patients with oral cGVHD, which can raise both ethical and logistical concerns. Based on our search up to this date there are two ongoing randomized clinical trials registered on centre and clinical trials.gov: Identifier: NCT01557517 and Identifier: NCT00887263. We expect the results of these trials likely to have an important impact on our confidence in the estimate of topical steroid effect.

The methodological limitations and inconsistency of the included clinical trials were adjudicated based on the following. (a) Three studies did not state the severity of the disease for participants at baseline [7, 11, 12]. (b) Two studies did not clearly report pain reduction which is very important outcome regarding the efficacy of the intervention [11, 12]. (c) All but one study [11] did not report the occurrence of relapse. (d) The sample size calculation was unclear for all but one study [7] and due to lack of clinical trials on chronic oGVHD, the sample size of this study was calculated based on studies of OLP. (e) Follow-up period was variant between studies and three studies [7, 10, 12] did not report clearly the follow-up. (f) For randomized clinical trials, randomization and allocation concealment were unclear [10] and blinding was unclear neither for the participant patients nor for outcome assessor [10]. Based on these findings one clinical trial was judged as high risk of bias [10] and one judged as low risk of bias [7].

In accordance was the review [4] which reported a shortage in clinical trials and low quality of the available evidence referring to the nature of the disease and ethical issues concerning the patients' condition. The review could not make recommendations regarding any topical intervention in oral cGVHD. Another study [14], a clinical survey, was conducted assessing clinical interventions for diagnosis and management of oral cGVHD. They reported that topical corticosteroids were the first line of treatment chosen by oral specialists.

Moreover, report from the International Consensus Conference on clinical practice in cGVHD stated that “current treatment recommendations rely on clinical experience of respected authorities or very small controlled trials rather than rigorously controlled trials.” In addition they denoted that topical treatment is of a special clinical value [1].

## 8. Conclusion

Based on the available evidence we concluded that there is moderate evidence supporting use of topical corticosteroid for management of oral cGVHD with minimal easily avoided side effects. According to level of evidence clobetasol followed by budesonide reported a promising clinical efficacy.Further research is likely to have an important impact on our confidence in the estimate of effect.

## Figures and Tables

**Table 1:** Study reports on the use of topical steroids in management of oral cGVHD.

Study	Author's name	Study	Design
1	Noce et al. [7]	Randomized double-blind clinical trial comparing clobetasol and dexamethasone for the topical treatment of symptomatic oral chronic graft versus host disease	Randomized clinical trial

2	Park et al. [8]	Comparison of budesonide and dexamethasone for local treatment of oral chronic graft versus host disease	Cohort (prospective)

3	Sari et al. [9]	The effect of budesonide mouthwash on oral chronic graft versus host disease	Cohort (retrospective)

4	Elad et al. [10]	Improvement in oral chronic graft versus host disease with the administration of effervescent tablets of topical budesonide—an open, randomized, multicenter study	Randomized clinical study (pilot study)

5	Wolff et al. [11]	Oral PUVA and topical steroids for treatment of oral manifestations of chronic graft versus host disease	Cohort (prospective)

6	Elad et al. [12]	Budesonide: a novel treatment for oral chronic graft versus host disease	Before and after clinical trial

**Table 2:** The evidence table including study design, interventions, outcomes, level of evidence, and grade of recommendation.

Study	Quality assessment		Number of patients	Drug properties				Clinical response (efficacy)				Safety %
	Level	Grade		Drug	Conc.	Dose		Average time for best results (D)	Average mOMRS reduction (%)	Patients with mOMRS improvement (%)	VAS red. (%)	Side effects %
						mL	t/d					
Clobetasol												
Noce et al. [7]	1b	A	14	Oral rinse	0.5	5	3	28	50	85	44.6	7

Dexamethasone												
Noce et al. [7]	1b	A	18	Oral rinse	0.01	5	3	28	50	33	29.7	5.5
Park et al. [8]	2b	B	24	Oral rinse	0.01	10	3-4	28	27.2	29.1	28.5	16.6
Wolff et al. [9]	4	C	16	Oral rinse	0.01	NR	NR	28	NR	68.7	NR	NR

Budesonide												
Park et al. [8]	2b	B	26	Oral rinse	0.03	5–10	3–6	28	50	53.8	37.5	19
Elad et al. [10]	2b	B	18	Oral rinse	0.03	10	2-3	45.5	70	70	70	50
Sari et al. [9]	4	C	12	Oral rinse	0.03	10	3	28	66.6	83.3	54.4	16.6
Elad et al. [12]	4	C	12	Oral rinse	0.06	5	2-3	54	UC	100	UC	8

*RCT*: randomized clinical trial, *pro-cohort*: prospective cohort, *Retro. Cohort*: retrospective cohort, *UC*: unclear, and *NR*: not reported.
